# Prediction of difficult tracheal intubations in thyroid surgery. Predictive value of neck circumference to thyromental distance ratio

**DOI:** 10.1371/journal.pone.0212976

**Published:** 2019-02-27

**Authors:** Alessandro De Cassai, Francesco Papaccio, Giorgia Betteto, Chiara Schiavolin, Maurizio Iacobone, Michele Carron

**Affiliations:** 1 Department of Medicine—DIMED, Section of Anesthesiology and Intensive Care, University of Padova, Padova, Italy; 2 Department of Surgery, Oncology and Gastroenterology, Endocrine Surgery Unit, University of Padua, Padua; IRCCS Policlinico S.Donato, ITALY

## Abstract

**Introduction:**

Difficult tracheal intubation (DTI) contributes to perioperative morbidity and mortality. There are conflicting study results about the most predictive DTI risk criteria in patients undergoing thyroid surgery.

**Materials and methods:**

We conducted a prospective observational study on 500 consecutive patients aged ≥18 years to identify predictors for DTI. Body weight, body mass index (BMI), inability to prognath, head movement, mouth opening, Mallampati score, neck circumference (NC), thyromental distance (TMD), neck circumference to thyromental distance ratio (NC/TMD), tracheal deviation apparent on chest x-ray, mediastinal goiter, histology and history of DTI were measured as possible predictors of DTI. Spearman's rank correlation test and multiple logistic regression analysis were performed.

**Results:**

DTI was observed in 9.6% of all patients. Compared with the group of patients without DTI, the group of patients with DTI had significantly greater median values for body weight, BMI, NC, NC/TMD, Mallampati score, el-Ganzouri score, incidence of mediastinal goiter, and had reduced TMD and mouth opening. Significant correlations between BMI ≥30 kg/m^2^ and the Mallampati score ≥3 (R = 0.124, p = 0.00541), Cormack-Lehane ≥3 (R = 0.128, p = 0.00409), NC ≥40 cm (R = 0.376, p<0.001), and NC/TMD ≥5 (R = 0.103, p = 0.0207) were found. The logistic regression analysis revealed that an NC ≥40 cm at the goiter level, but not an NC/TMD ratio ≥5, was the strongest predictor of DTI (p<0.001). The area under the receiver operating characteristic curve for NC/TMD was better than the curve for NC. The sensitivity and specificity of NC/TMD were also greater, compared with NC. An NC of 40.00 cm and an NC/TMD of 5.85 were the estimated cut-off points.

**Discussion:**

This study found that NC was a strong predictor of DTI. The results also suggested that NC/TMD could be used as a measure to stratify the risk of DTI in patients undergoing thyroid surgery.

## Introduction

Difficult tracheal intubation (DTI) contributes to perioperative morbidity and mortality [1,2]. Identification of DTI risk criteria is essential while planning the best way to proceed with anesthesia [1]. There are conflicting study results about the most predictive DTI risk criteria in patients undergoing thyroid surgery [3–7]. Neck circumference (NC) seems to be an important predictor for DTI in this population of patients [5–7]. A study of a large cohort of patients undergoing thyroidectomy found that the size of the neck (circumference and length) is one of most important independent predictors of DTI in thyroid surgery [5]. Cut-off values for neck width have not been established. The relationships between neck circumference and length as predictors of DTI have also not been evaluated [5]. One study of a population of patients with obesity found that the NC to thyromental distance (NC/TMD) ratio is a better method for predicting DTI than other established indices [8].

The aim of the study was to identify predictors for DTI and to examine the relative value of NC versus NC/TMD for predicting DTI in patients undergoing thyroid surgery.

## Materials and methods

### Ethical statement

The study protocol was approved by the Ethics Committee for Clinical Research of the Padova Medical Hospital (Reference number: 4133/AO/17) on April 06^th^, 2017. The study protocol was in accordance with the 1964 Declaration of Helsinki and its later amendments. Informed written consent was obtained from each participant included in the study. This is a non-interventional (observational) clinical study, based on routine care, for which the registration at public registry is not necessary at or before the time of first patient enrollment. However, we decided for a voluntary submission of our study at ClinicalTrials.gov (reference number: NCT03578601) that was concluded on July 06^th^, 2018 due to difficulties encountered from account creation to final record release at Clinicaltrials.gov. The study started on September 01^st^, 2017 and ended on September 17^th^, 2018. The authors confirm that all ongoing and related trials for this intervention are registered.

### Population

**Fig 1:**
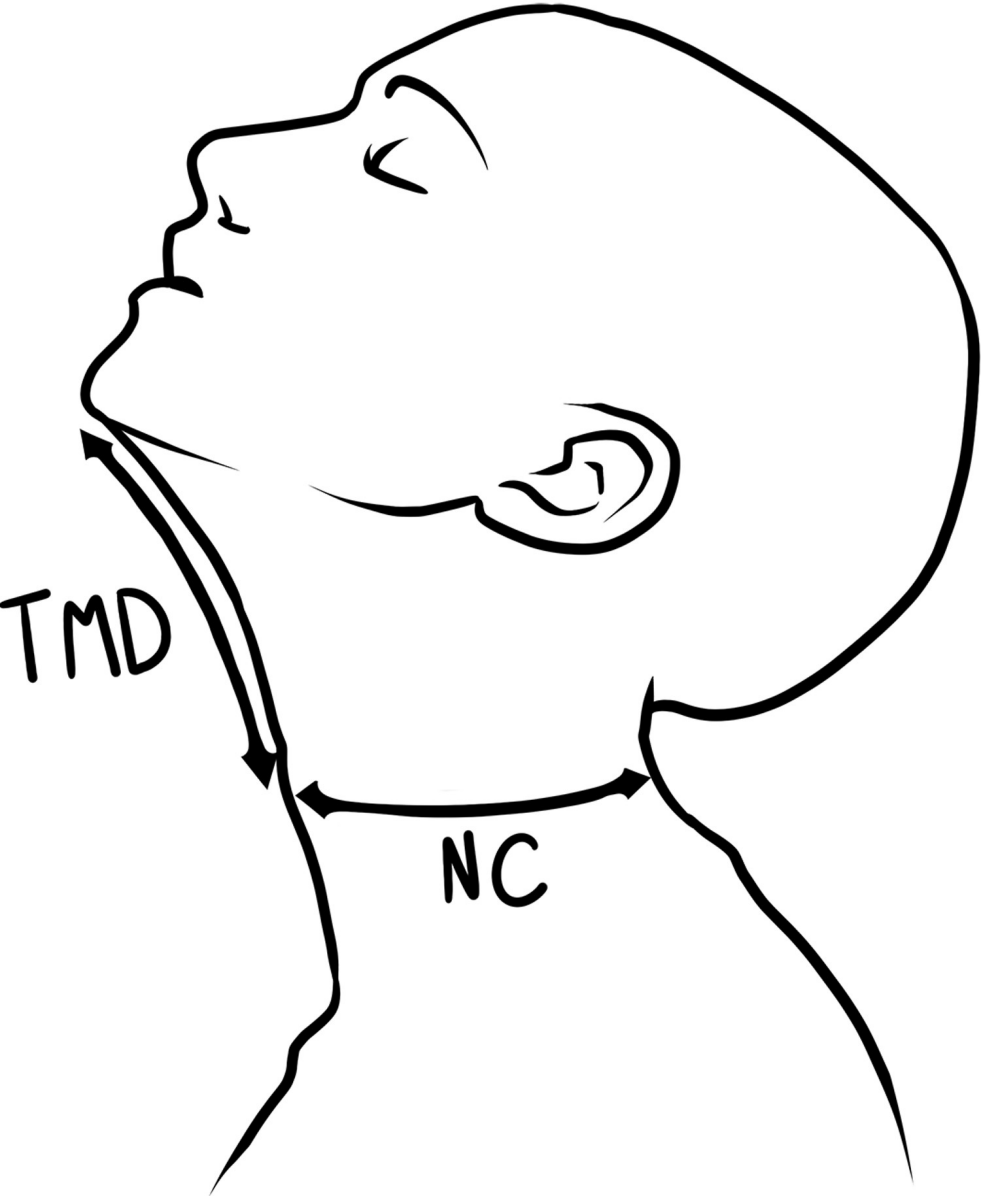
NC measurement and TMD measurement: NC measurement and TMD measurement.

**Fig 2:**
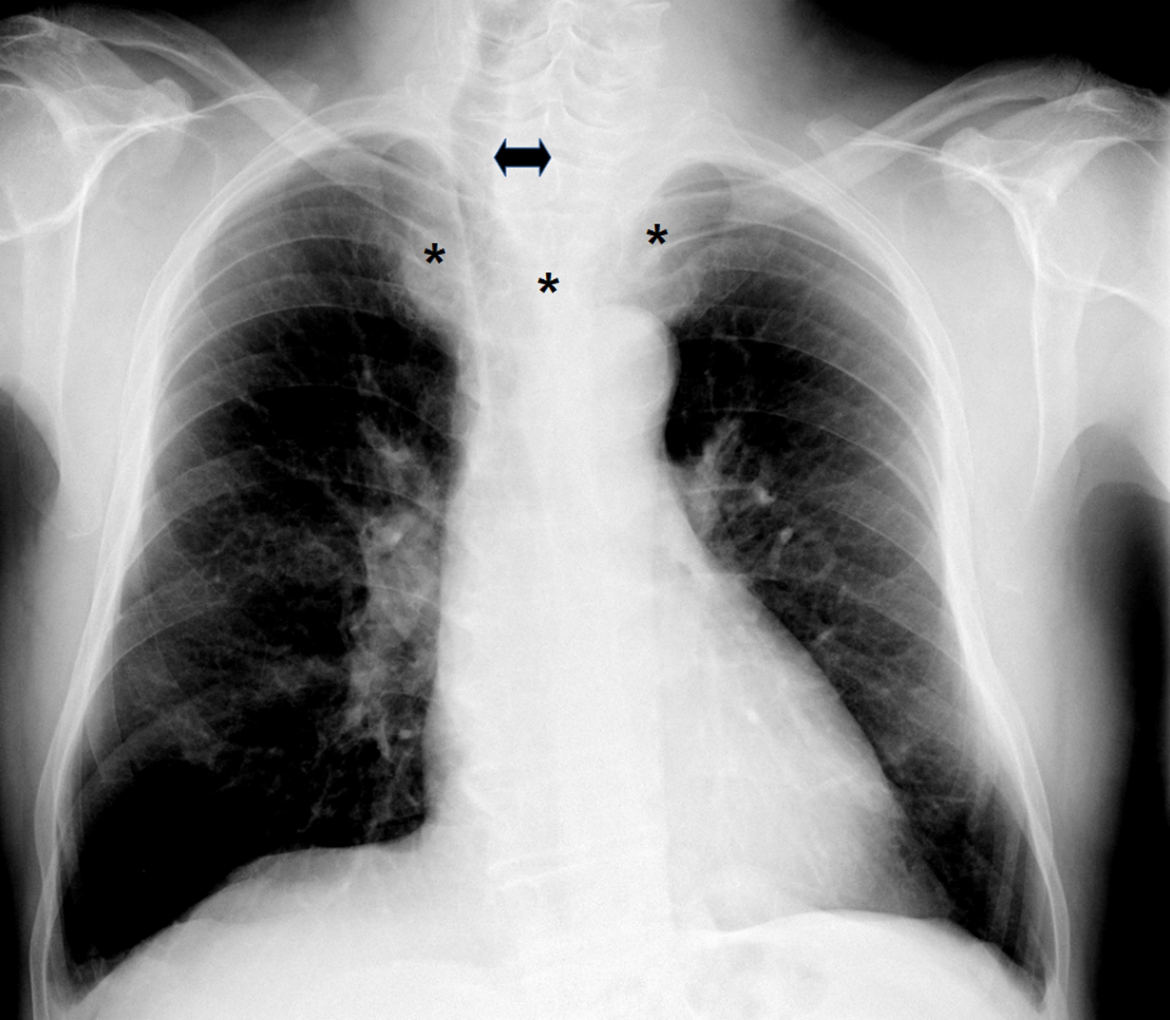
Mediastinal goiter and tracheal deviation at CXR: Asterisks: Mediastinal goiter shadow, arrow: Tracheal deviation.

Patients (≥ 18 years of age) scheduled for thyroid surgery at University Medical Hospital of Padova (Italy) were considered. Patients were recruited consecutively until the sample size was achieved. Patients with one or more anatomical abnormalities, pathologies, and previous surgery suggesting use of a none-standard approach or fiberoptic awake intubation were excluded from the study [9]. Body weight, body mass index (BMI), inability to prognath, head movement, mouth opening, Mallampati score, NC, TMD (Fig 1), NC/TMD, tracheal deviation apparent on chest x-ray (radiograph) (CXR), mediastinal goiter (Fig 2), histology and history of DTI were measured as possible predictors of DTI. The analysis included dichotomization and stratification of continuous and non-continuous variables into “risk” categories: BMI (<30 kg/m2, ≥30 kg/m2) [10]; inability to prognath (yes/no); head movement (≤90°, >90°); mouth opening (<4 cm, ≥4 cm); Mallampati score: <III, ≥III; NC (<40 cm, ≥40 cm) [11]; TMD (≤6.5 cm; >6.5 cm); NC/TMD (<5; ≥5) [8]; tracheal deviation at CXR (yes/no); mediastinal goiter (yes/no) histology (benign/malignant); history of DTI (yes/no) [12].

DTI was defined as maneuver performed with a correct head position and external laryngeal manipulation resulting in: a) difficult laryngoscopy (Cormack-Lehane grade III or IV); b) more than one intubation attempt; c) needing of no standard devices and/ or procedures; d) withdrawal and procedure re-planning [9].

Preoperative airway evaluation was performed by the attending anesthesiologist not involved in the perioperative part of this study.

All anesthesiologists followed the national [9] and the international [13] guidelines for management of unanticipated DTI in adults.

### Endpoints

The estimation of the prevalence of DTI in a population of patients undergoing thyroid surgery and the identification of predictors for DTI were the main endpoints of the study. The examination of NC/TMD for predicting DTI in patients undergoing thyroid surgery was the secondary endpoint of the study.

### Statistical analysis

The estimates of study power and sample size were based on an assumption of a difference of 4% in the incidence of difficult intubation between the general population of patients undergoing surgery [1] and the population of patients undergoing thyroid surgery [4], We use an α-value of 5%, a power (1-β error) of 95%, a drop out percentage of 5%. The results of the analysis indicated that a sample size of 515 patients was necessary to estimate the prevalence of DTI in a population of patients undergoing thyroid surgery.

The data for each continuous variable were analyzed for a normal distribution using the Kolmogorov-Smirnov test. Results for continuous variables with normal distributions were expressed as mean (standard deviation) values; those with non-normal distributions were expressed as median (interquartile range) values. Analysis of data with a normal or a non-normal distribution was performed using the two-tail Student’s t-test and the Mann-Whitney U test, respectively. The results for analyses of categorical variables were reported as numbers (percentages) and were compared between groups using Chi-square tests.

To determine the strength and direction of association between two variables, we used the Bravais-Pearson’s correlation test for variables with a normal distribution and Spearman's rank correlation test for variables that did not meet the assumptions of a normal distribution.

To determine the relationships between the dependent categorical variable (i.e., DTI) and one or more independent categorical variables (i.e., DTI predictors), we performed a multiple logistic regression analysis to calculate odds ratios (ORs) with 95% confidence intervals (CIs). The presence of multicollinearity was detected using variance inflation factors. Using the Akaike information criterion, we performed backward and forward stepwise regression to select the best model.

The area under the receiver operating characteristic (ROC) curve was used to examine the predictive performance of DTI predictors (i.e. NC and NC/TMD). The sensitivity and specificity plots were used to determine the cut-off points for DTI prediction [8,11].

All statistical analyses were performed using R version 3.4.0 (2017-04-21). *P-*values <0.05 were considered to indicate a statistically significant result.

## Results

**Fig 3:**
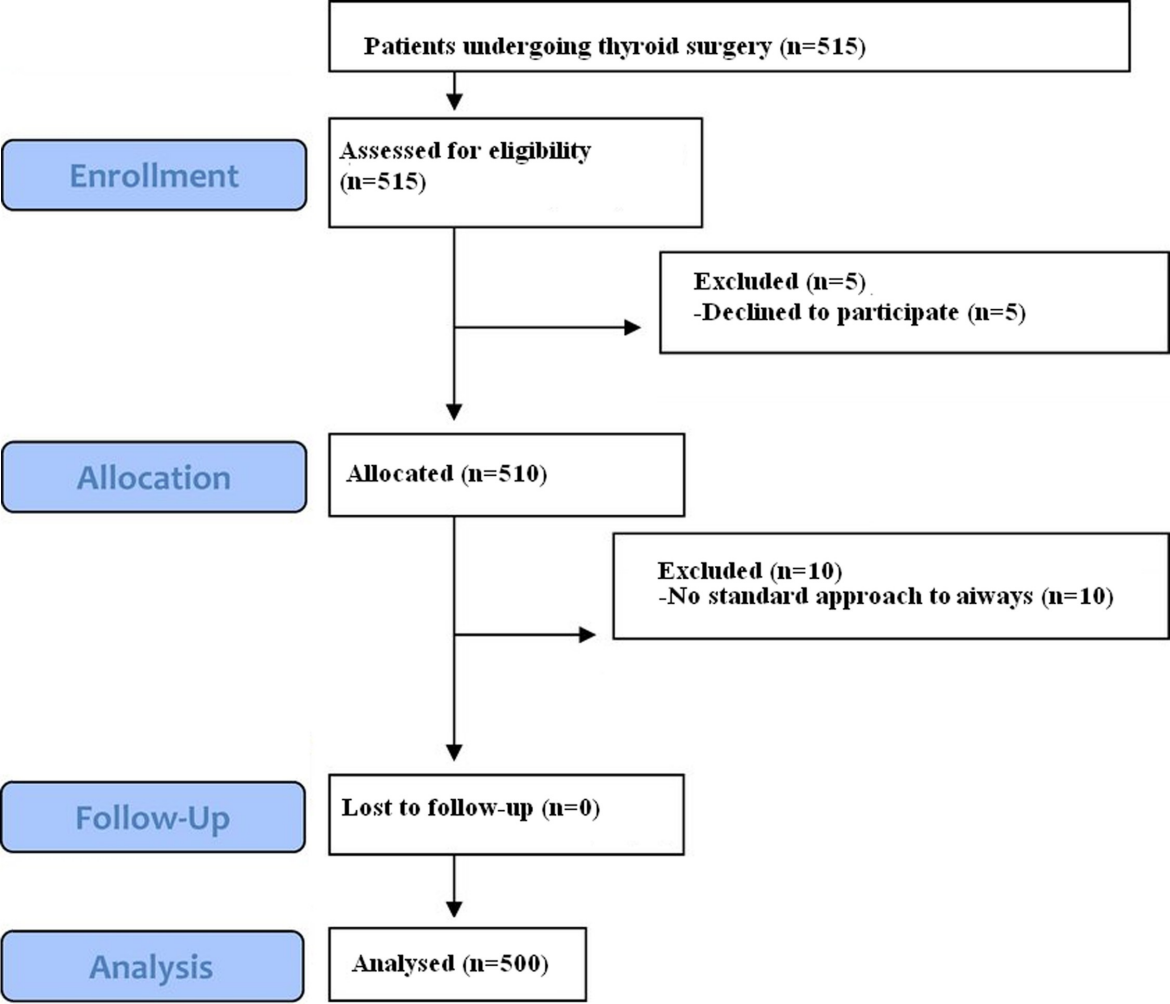
TREND flowchart.

A total of 515 patients were enrolled in this study from 1^st^ September 2017 to 17^th^ September 2018. Fifteen patients were excluded before the final analysis was performed (5 patients refused to participate, 10 patients had no standard approach to the airway available). The remaining 500 patients completed the study (Fig 3). The data from these patients were included in the analysis.

The results for the population’s demographic characteristics are presented in Table 1. DTI occurred in 9.6% of all patients. Airway control was successfully achieved using videolaryngoscopy in 33.3%, a Frova catheter in 31.2%, fiberoptic intubation via i-gel supraglottic airway in 18.7%, and blind intubation in 16.6% of the patients. There were no complications.

**Table 1:** Characteristics patients.

Variable		Patients		p-value
		No DTI (n = 452)	DTI (n = 48)	
Sex	Male	100 (22.1)	14 (29.2)	0.279
	Female	352 (77.9)	34 (70.8)	
Age (y)		53.0 [43.00, 64.00]	46.61 [49.75, 62.25]	0.215
Weight (kg)		67.0 [60.0, 78.2]	75.0 [68.7, 85.0]	<0.001
Height (m)		1.65 [1.60, 1.72]	1.68 [1.62, 1.73]	0.487
BMI (kg/m^2^)		24.2 [22.0, 27.2]	27.3 [24.4, 30.0]	<0.001
BMI ≥30 kg/m^2^		55 (12.2)	13 (27.1)	0.008
NC (cm)		36.5 [34.0, 40.0]	41 [40.0, 42.2]	<0.001
NC ≥40 cm		135 [29.9]	37 [77.1]	<0.001
TMD (cm)		8.0 [6.5, 9.0]	7.0 [6.0, 8.0]	0.001
TMD ≤6.5		93 (20.6)	18 (37.5)	0.010
NC/TMD		4.7 [3.9, 5.7]	6.0 [4.9, 7.0]	<0.001
NC/TMD ≥5		265 (58.6)	39 (81.29	0.002
Mouth opening <4 cm		38 (8.4)	13 (27.1)	<0.001
Inability to prognath		30 (6.69	3 (6.2)	1.0
Neck movement ≤90°		84 (18.69	14 (29.29	0.086
Mallampati score		1.0 [1.0, 2.0]	2.0 [2.0, 3.0]	<0.001
Mallampati score ≥III		47 (10.4)	18 (37.5)	<0.001
Past difficult intubation		2 (0.4)	4 (8.3)	0.001
el-Ganzouri score		1.0 [0.0, 2.0]	2.0 [1.0, 3.2]	<0.001
el-Ganzouri score ≥4		35 (7.7)	13 (27.1)	<0.001
Tracheal deviation at CXR		91 (20.1)	13 (27.1)	0.264
Mediastinal goiter		44 (9.7)	13 (27.1)	0.001
Malignancy at HP		118 (26.1)	15 (31.2)	0.492

The results for continuous variables are expressed as median (interquartile range) values. Categorical variables are reported as numbers (percentages). ***Abbreviations*:** DTI: difficult tracheal intubation; BMI: body mass index; NC: neck circumference; TMD: thyromental distance; NC/TMD: NC to thyromental distance ratio; CXR: chest x-ray (radiograph); HP: histopathology

Compared with the group of patients without DTI, the group of patients with DTI had significantly greater median values for body weight, BMI, NC, NC/TMD, Mallampati score, el-Ganzouri score, incidence of mediastinal goiter, and had reduced TMD and mouth opening Table 1.

The correlations between BMI ≥30 kg/m^2^ and the Mallampati score ≥3 (R = 0.124, p = 0.00541), Cormack-Lehane ≥3 (R = 0.128, p = 0.00409), NC ≥40 cm (R = 0.376, p<0.001), and NC/TMD ≥5 (R = 0.103, p = 0.0207) were statistically significant. The correlations between the presence of benign goiter and tracheal deviation on CXR (R = 0.108, p = 0.0159) and between benign goiter and mediastinal goiter (R = 0.116, p = 0.00928) were statistically significant.

The logistic regression analysis revealed that an NC ≥40 cm at the goiter level, but not an NC/TMD ratio ≥5, was the strongest predictor of DTI (p<0.001). Multicollinearity was not detected using variance inflation factors Table 2.

**Table 2:** Multiple linear regression analysis to explain the relationship between DTI and variables considered.

Variable	Regression Model				Fitted regression model		
	VIF	OR	95%CI	*P*-value	OR	95%CI	*P*-value
Sex (male)	1.268	1.56	0.68–3.52	0.288			
BMI ≥30 kg/m^2^	1.190	1.23	0.53–2.88	0.626			
DTM ≤6.5	1.367	1.24	0.53–2.87	0.622			
NC/TMD	1.191	1.52	0.62–3.66	0.354			
Mouth opening <4 cm	1.210	2.67	1.06–6.74	0.037	2.97	1.24–7.10	0.014
Inability to prognath	1.248	0.39	0.08–2.12	0.244			
Neck movement ≤90°	1.191	0.85	0.37–1.96	0.706			
NC ≥40 cm	1.413	7.25	3.01–17.5	<0.001	6.68	3.12–14.3	<0.001
Mallampati ≥III	1.222	2.23	1.03–6.01	0.042	2.19	1.03–4.76	0.047
Past difficult intubation	1.231	20.9	1.91–229	0.012	12.6	1.55–103	0.017
Tracheal deviation at CXR	1.171	0.65	0.27–1.54	0.329			
Mediastinal goiter	1.246	3.03	1.19–7.67	0.019	2.66	1.16–6.10	0.021
Malignancy at HP	1.157	1.95	0.89–4.27	0.095	1.73	0.82–3.61	0.144

The results for continuous variables are expressed as median (interquartile range) values. Categorical variables are reported as numbers (percentages). ***Abbreviations*:** DTI: difficult tracheal intubation; BMI: body mass index; NC: neck circumference; TMD: thyromental distance; NC/TMD: NC to thyromental distance ratio; CXR: chest x-ray (radiograph); HP: histopathology

**Fig 4:**
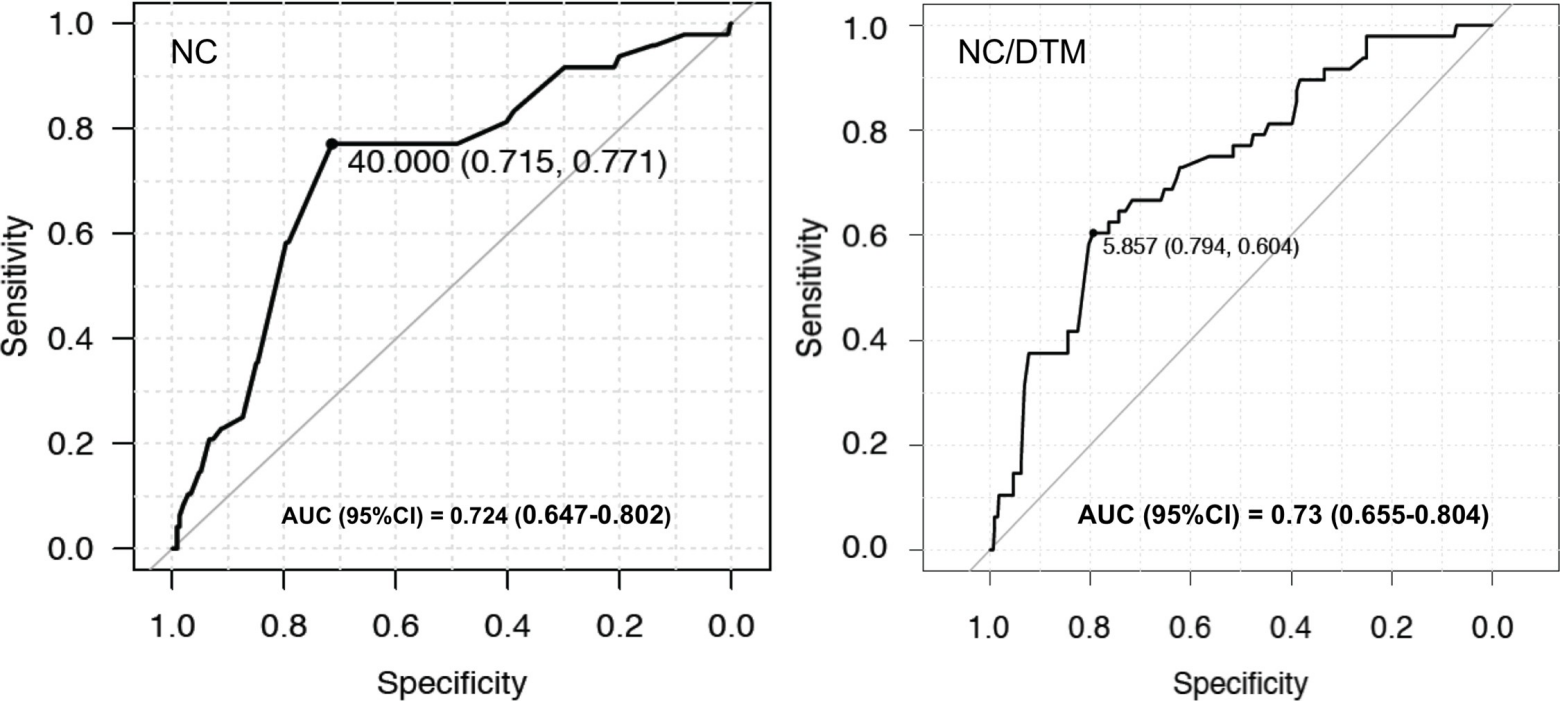
Receiver operating characteristic (ROC) curves with area under ROC curve (AUC) values (95% confidence intervals) for neck circumference (NC) measurement and the ratio of the NC to thyromental distance (NC/TMD).

The ROC curve for NC/TMD was better than the curve for NC (Fig 4).

**Fig 5:**
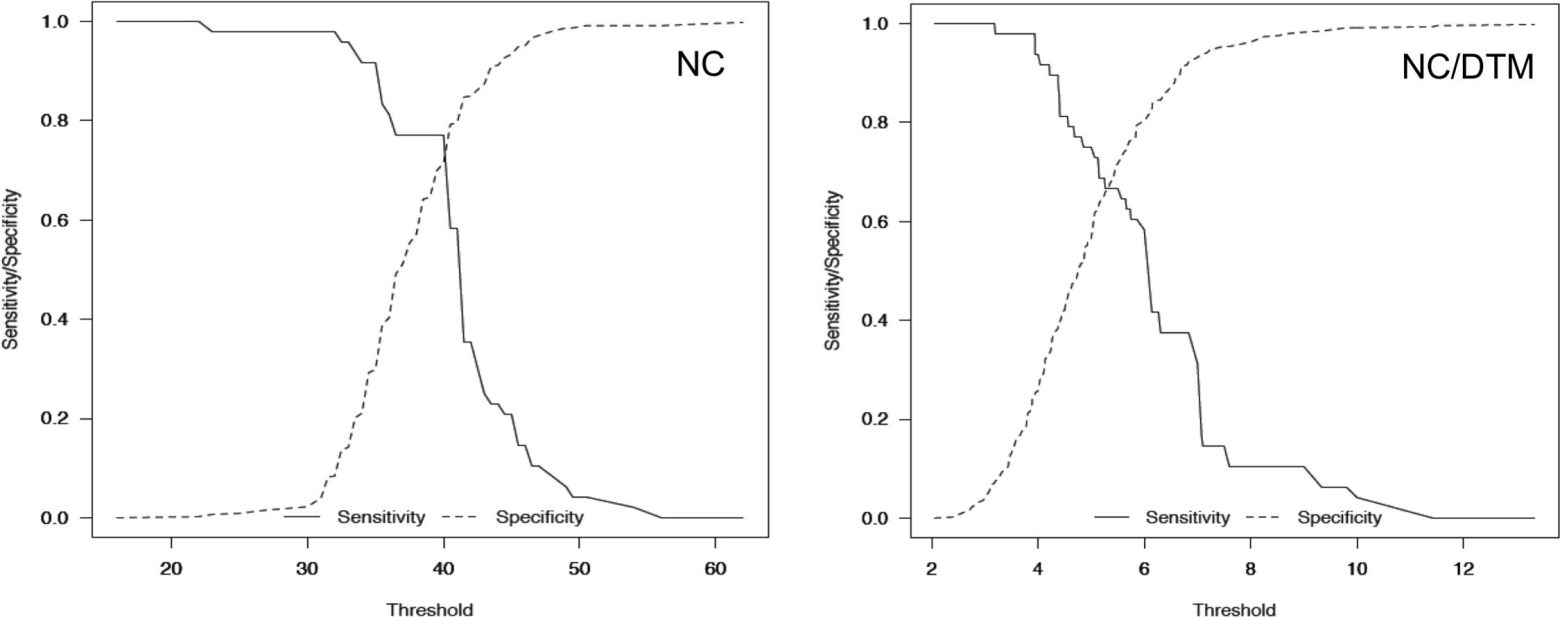
Values for sensitivity and specificity for neck circumference (NC) measurement with a 40-cm cut-off value and ratio of the NC to thyromental distance (NC/TMD) test with a cut-off value of 5.85.

The sensitivity and specificity of NC/TMD were also greater, compared with NC. An NC of 40.00 cm and an NC/TMD of 5.85 were the estimated cut-off points (Fig 5).

## Discussion

This study revealed a high incidence of DTI in this population of patients undergoing thyroid surgery. The results indicated that NC was a strong predictor, but that the NC/TMD ratio was the best assessment to use to predict the risk of DTI.

A meta-analysis found that the overall incidence of DTI in patients with no airway pathology is 6.2% for normal patients (excluding obstetric patients and patients with obesity) [14]. The incidence of DTI in populations of patients undergoing thyroid surgery varies. Some studies found similar values for incidence of DTI (5.3% to 6.8%) [3,5,15–18]. Other studies found higher values (10% to 13.6%) [4,6,7,19,20]. Our population of patients undergoing thyroid surgery had an increased incidence of DTI (9.6%).

Prediction of DTI has high clinical importance, and previous studies have evaluated risk factors for DTI in patients undergoing thyroid surgery [3,5–7,19,20]. Increased age, a high Mallampati score, a Cormack Grade score III or IV, a reduced mouth opening (<4.4 cm), a cancerous goiter and a tracheal stenosis (≥30%) are independent predictors for DTI [3,7,20]. The role of NC and TMD have also been investigated [5,6,18]. Two studies identified NC as an independent predictor for DTI in thyroid surgery without estimating cut-off values for risk categories [5,6]. Only one study found that TMD (<6.5 cm) was associated with an increased risk (OR [95%CI]: 2.326 [1.014–5.338], p = 0.046) of DTI in thyroid surgery patients [18]. Our study found that both NC and TMD differed significantly in DTI compared with patients without DTI, but only NC was an independent predictor of DTI in thyroid surgery patients. Kalezić et al. suggested that an increased NC and an associated reduced NC length could affect the risk of DTI in thyroid surgery patients [5]. The NC/TMD ratio was developed by Kim et al. based on the assumption that patients with obesity who had both a large neck circumference and a short neck length might be more difficult to intubate than patients with only a large neck circumference or a short neck [8]. Obesity and thyroid gland disease in the same patient increase NC and NC/TMD. Obesity contributes by increasing the amount of neck soft tissue [8,21]. A study using magnetic resonance imaging found that the greater proportion of increased soft tissue is due to increased fat, but this change is more likely to be present in women with obesity than in men with obesity [22]. However, the increased soft tissue sustained by fat deposition in the neck does not completely account for the relatively poorer ability to view the vocal cords during direct laryngoscopy. The increased amount of pre-tracheal neck soft tissue most likely impairs laryngoscopy by reducing the anterior mobility of pharyngeal structures [21]. Brodsky et al. found that a larger NC is associated with a higher Mallampati score (p = 0.0029) and Grade 3 views during laryngoscopy (p = 0.0375) [11]. Similarly, we found that obesity was associated with higher Mallampati and Cormack-Lehane scores at laryngoscopy in patients undergoing thyroid surgery with DTI, compared with those with no DTI. NC, but not TMD, may be an indication of the amount of neck soft tissue. TMD is a surrogate for inadequate head extension, rather than the size of the submandibular space [23]. However, NC/TMD might represent the distribution of fat in the neck better than NC alone [8]. Our study found that NC/TMD was not an independent risk factor for DTI. However, the results of the ROC curve analysis indicated that an NC/TMD ≥5.85 had better predictive performance than NC. This NC/TMD value differs from the value of NC/TMD ≥5 proposed by Kim for patients with obesity [8].

Thyroid gland disease also contributes to NC enlargement and DTI. Thyroid gland volume is not correlated with DTI [17], However, goiter (OR [95%CI]: 2.513 [1.139–5.542], p = 0.022) and thyroid weight (OR [95%CI]: 2.232 [1.075–4.636], p = 0.031) are independent risk factors for DTI in thyroid surgery patients [18]. Consistent with our results, invasion and infiltration of adjacent tissues by thyroid gland disease, more than expansion of thyroid gland volume, affects the neck and predicts DTI in patients undergoing thyroid surgery [17].

This study had some limitations. First, we did not use an Intubation Difficult Scale to stratify the risk of DTI [24]. Use of this scale would have increased the comparability of the results of this study to the results of other studies. Second, no imaging evaluation (e.g., ultrasound, computed tomography, magnetic resonance imaging scans) other than CRX was used to investigate the neck soft tissue, volume and structure of the gland, and the surrounding structures. CRX was the only imaging method used for all patients undergoing thyroid surgery. Third, sensitivity and specificity was assessed for single measures. Evaluation of combinations of measures might further increase the sensitivity for prediction of DTI. Fourth, examinations were performed by the study team; they were very familiar with all tests performed. The results in clinical practice might differ, especially when rarely employed measures are used by less experienced providers.

## Conclusions

Our study confirms the increased risk of DTI in patients undergoing thyroid surgery. Several predictors should be considered in clinical practice, such as reduced mouth opening, a high Mallampati score, an increased NC, a history of difficult intubation and a mediastinal goiter. This study found that increased NC ≥40 cm was the strongest predictor of DTI. The results also suggested that NC/TMD ≥5.85 could be used as a measure to stratify the risk of DTI in patients undergoing thyroid surgery. Measurement of NC and NC/TMD should be considered during preoperative evaluation in order to predict DTI in patients undergoing thyroid surgery.

## Supporting information

S1 Checklist Trend checklist for the “Prediction of difficult tracheal intubations in thyroid surgery. Predictive value of neck circumference to thyromental distance ratio” study.
(PDF)

S1 Protocol Original protocol for the “Prediction of difficult tracheal intubations in thyroid surgery. Predictive value of neck circumference to thyromental distance ratio” study in Italian language.
(PDF)

S2 Protocol English translation of original protocol for the “Prediction of difficult tracheal intubations in thyroid surgery. Predictive value of neck circumference to thyromental distance ratio” study.
(PDF)

S1 Database Study database. (XLSX)
