# Supplementary material for: Prediction of difficult tracheal intubations in thyroid surgery. Predictive value of neck circumference to thyromental distance ratio
Source: PLoS One. 2019 Feb 27;14(2):e0212976. doi: 10.1371/journal.pone.0212976 (PMC6392301; doi:10.1371/journal.pone.0212976)
Supplement: S1 Protocol — Predictive value of neck circumference to thyromental distance ratio” study in Italian language. (PDF) [file pone.0212976.s002.pdf]

Studio osservazionale prospettico monocentrico: Identificazione di parametri predittivi di intubazione difficile in pazienti da sottoporre a tiroidectomia

Vers. 1.4

Protocol Version Date: 09/02/2017

Contenuti

1. Informazioni generali
1. Background
1.1. Definizioni
1.2. Dimensione del problema
1.3. Previsione di difficoltà
1.4. Previsione di difficoltà e patologia tiroidea
1.5. Razionale dello studio
2. Obiettivi dello studio e ipotesi
   2.1. Obiettivo primario e Ipotesi
   2.2. Obiettivo secondario
3. Criteri di inclusione esclusione
3.1. Arruolamento
4. Materiali e metodi
4.1. Raccolta dati
4.2. Statistica
4.2.1. Numerosità campionaria
4.2.2. Analisi statistica
5. Rischi e benefici per il paziente
6. Costi addizionali
7. Bibliografia

1. Informazioni generali

Titolo dello studio

Identificazione di parametri predittivi di intubazione difficile in pazienti da sottoporre a tiroidectomia

Protocol Version 1.4 – 09 Febbraio 2017

2. Background

2.1 Definizioni

Per intubazione difficile[1], si intende una procedura che sia stata caratterizzata da una laringoscopia difficile o abbia richiesto almeno quattro tentativi o più di cinque minuti per la sua esecuzione indipendentemente dal grado di esperienza dell'anestesista.

Per laringoscopia difficile si intende l'impossibilità ad evidenziare la glottide con il laringoscopio convenzionale a lama curva. Essa corrisponde ai gradi 3 e 4 della classificazione di Cormack e Lehane (Allegato 1) nei quali si riesce ad evidenziare rispettivamente la sola epiglottide o la sola lingua.

2.2 Dimensione del problema

I dati di incidenza riportati dalla letteratura sul controllo difficile delle vie aeree sono spesso contradittori perchè hanno risentito nel passato della mancanza di univoche definizioni. Il 30% degli incidenti totalmente riferibili all'anestesia è sostenuto da difficoltà di controllo delle vie aeree, il 70% di questi incidenti ha comportato morte o danno cerebrale permanente. L'incidenza di intubazione difficile nella popolazione generale è compresa tra lo 0.5% e il 20%

2.3 Previsione di difficoltà

La previsione di difficoltà si basa sull'esame obiettivo del paziente con valutazione dei seguenti parametri:

- misura distanza interdentaria
- test di Mallampati (Allegato 2)
- visibilità strutture faringee
- misura distanza mento-tiroide
- misura prognatismo mascellare
- motilità globale del collo

La previsione di difficoltà o di impossibilità a intubare si basa sulla deviazione modesta di più parametri o sulla grave alterazione di un singolo parametro. In particolare la letteratura [1] ritiene che diano certezza di elevata difficoltà i seguenti parametri

- distanza interdentaria < 2 cm
- grave prognatismo mascellare
- test di mallampati 4
- collo fisso in estensione

- esiti cicatriziali o attinici gravi a carico del pavimento linguale

2.3 Previsione di difficoltà e patologia tiroidea

I pazienti con patologia tiroidea rappresentano una classe particolare di pazienti per quanto riguarda l'intubazione tracheale per la stretta contiguità anatomica tra tiroide e trachea: appare chiaro quindi come un ingrandimento della ghiandola tiroidea possa causare dislocamento e compressione delle vie aeree.

Sono stati già condotti alcuni studi riguardanti vie aeree e patologia tiroidea [2,3,4,5,6].La percentuale di intubazione difficile è risultata variabile dallo 0% [4] al 12,9%[5] I criteri analizzati in letteratura sono: distanza interdentaria, test di mallampati, distanza tireomentoniera, presenza di mento sfuggente, mobilità del collo, peso, età, presenza di deviazione tracheale all'RX, presenza di stenosi tracheale all'RX, carattere maligno della patologia tiroidea, diametro del collo, presenza di gozzo mediastinico, volume ghiandolare.

Nessuno degli studi presenti in letteratura ha analizzato tutte queste caratteristiche, ma ognuno si è soffermato su caratteristiche diverse, lasciando quindi la mancanza di uno studio che analizzi nella globalità queste caratteristiche in questa classe di pazienti.

Inoltre le conclusioni sono contrastanti; caratteristiche quali apertura della bocca, mallampati, distanza tireomentoniera, mento sfuggente, mobilità del collo, deviazione tracheale, natura maligna della patologia tiroidea e stenosi tracheale sono risultati significativi in alcuni studi e non significativi in altri; tale rilievo può essere giustificato dalla bassa numerosità degli studi, infatti lo studio di maggiore numerosità è di 326 pazienti [3] e quello a più bassa numerosità di 50 pazienti [4].

2.5 Razionale dello studio

La principale strategia per evitare incidenti di controllo delle vie aeree è la pianificazione e la previsione di possibili difficoltà[7,8].

Sebbene esistano diversi score sulla previsione di difficoltà per la intubazione difficile poco esiste riguardante la classe di pazienti con gozzo[2,3,4,5,6].

Il razionale è quindi quello di valutare se i criteri predittivi di intubazione difficile già validati per la popolazione sono validi anche per la popolazione affetta da patologia tiroidea, inoltre valutare se esistono caratteristiche peculiari di questa popolazione (diametro del collo, dislocamento tracheale, gozzo mediastinico) che possano aiutare nel predire la difficoltà di intubazione.

Lo studio si propone quindi di superare le criticità degli studi precedenti sia per quanto riguarda la numerosità campionaria, sia per quanto riguarda l'analisi complessiva dei parametri riguardanti

l'intubazione difficile senza soffermarsi, a differenza degli studi precedenti, soltanto su alcuni di essi.

3. Obiettivi dello studio e ipotesi

3.1. Obiettivo primario e ipotesi

Valutazione incidenza intubazione difficile nella popolazione con patologia tiroidea rispetto ai dati forniti dalla letteratura nella popolazione generale

3.2. Obiettivo secondario

Identificazione di parametri o pattern di parametri predittivi di intubazione difficile durante tiroidectomia.

Valutazione del rapporto NC e TMD nell'intubazione difficile in paziente da sottoporre a chirurgia tiroidea

4. Criteri di inclusione/esclusione

Criteri inclusione

- ✓ Programma chirurgico di tiroidectomia totale o parziale
- ✓ Età > 18 anni

Criteri esclusione

- ✓ Età < 18 anni
- ✓ Intubazione difficile prevista

4.1 Arruolamento

Questo studio osservazionale prospettico sarà condotto nelle sale operatorie del primo e secondo piano del policlinico dell'Azienda Ospedaliera di Padova.

Verranno arruolati tutti i pazienti che necessitano di intervento di tiroidectomia.

Nessuna decisione clinica verrà fatta in base ai dati raccolti per il presente studio. Infatti durante lo studio verranno registrate soltanto informazioni che già normalmente vengono rilevate durante una visita anestesiologica di routine.

5. Materiali e metodi

Durante la visita anestesiologica di routine previa raccolta del consenso infromato verranno registrati i seguenti parametri sul foglio di raccolta dati.

- Apertura bocca (cm)
- Distanza tireomentoniera (cm)
- Test di Mallampati (Allegato 2)
- Mobilità del collo (<80°; 80-90°; >90°)
- Inabilità di proganre
- Peso corporeo (kg)
- Storia di pregressa intubazione difficile
- Deviazione tracheale all’RX torace
- Cinconferenza del gozzo (cm)
- Presenza di gozzo mediastinico
- Natura benigna o maligna della patologia tiroidea

Il giorno dell’intervento chirurgico dopo l’intubazione oro tracheale verrà segnalato sul foglio di raccolta dati la presenza o meno di difficoltà nella manovra; in particolare:

- Scala di Cormack (Allegato 1)
- Numero di tentativi necessari per eseguire con successo l’intubazione
- Tempo trascorso dall’induzione di anestesia generale e intubazione con successo
- Se sono stati utilizzati presidi avanzati per le vie aeree e quali

5.1 Raccolta dati

I dati saranno raccolti in una scheda raccolta dati. Ai pazienti verrà attribuito un codice numerico per facilitare la successiva elaborazione; i dati saranno conservati in forma anonimizzata.

5.2 Statistica

5.2.1 Numerosità Campionaria

Un campione di 500 pazienti consente di stimare la prevalenza di intubazione difficile, supposta pari al 10% [2,3], con un intervallo di fiducia bilaterale di livello 95% di precisione ±3%).numero che sarà possibile raggiungere in tre anni.,

5.2.2 Analisi Statistica

Le stime della potenza dello studio e della dimensione del campione sono basate su un'ipotesi di una differenza del 4% nell'incidenza di intubazione difficile tra la popolazione generale di pazienti e la popolazione di pazienti sottoposti a chirurgia tiroidea, usiamo un valore α del 5%, un potere del 95%, una percentuale di dropout del 5%. I risultati dell'analisi hanno indicato che un campione di 515 pazienti sia necessaria per stimare la prevalenza di difficoltà di intubazione difficile in una popolazione di pazienti sottoposti a chirurgia della tiroide.
I dati per ciascuna variabile continua saranno analizzati per una distribuzione normale utilizzando il Test di Kolmogorov-Smirnov. Saranno spressi i risultati per variabili continue con distribuzioni normali come valori medi (deviazione standard); quelli con distribuzioni non normali sarannoi espressi come mediana e intervallo interquartile. Sarò eseguita l'analisi dei dati con una distribuzione normale o non normale utilizzando il test t di Student a due code e il test U di Mann-Whitney, rispettivamente. I risultati per le analisi di variabili categoriali sono stati riportati come numeri (percentuali) e sono stati confrontati tra gruppi che usano test chi-quadrato.
Per determinare la forza e la direzione di associazione tra due variabili, sarà usato il Test di correlazione di Bravais-Pearson per variabili con distribuzione normale e rango di Spearman test di correlazione per variabili che non soddisfano le ipotesi di una distribuzione normale.
Per determinare le relazioni tra la variabile categorica dipendente (es. DTI) e una o più variabili categoriali indipendenti (cioè predittori DTI), sarà eseguito una logistic regression per calcolare gli odds ratio (OR) con intervalli di confidenza al 95% (IC). La presenza di multicollinearità è stata rilevata utilizzando fattori di inflazione di varianza. Tutte le analisi statistiche saranno eseguite utilizzando R versione 3.4.0 (2017-04-21). I valori di P<; 0.05 saranno considerato per indicare un risultato statisticamente significativo.

6. Rischi e benefici per il paziente

Lo studio è prospettico osservazionale. Nessuna decisione clinica verrà presa sulla base dei dati raccolti. Non ci sono benefici diretti nè rischi aggiunti a quelli di una procedura clinica standardizzata.

7. Costi addizionali

Lo studio è prospettico osservazionale senza costi aggiuntivi.

8. Limiti dello studio

Il principale limite dello studio è la sua natura monocentrica.

8.Bibliografia

1-Raccomandazioni per il controllo delle vie aeree e la gestione delle difficoltà. Gruppo di Studio SIAARTI Coordinatrice Flavia Petrini Minerva Anestesiologica 2005; 71: 617-67

2-Bouaggad A et al, Prediction of difficult tracheal intubation in thyroid surgery. Anesthesia & Analgesia: August 2004 - Volume 99 - Issue 2 - pp 603-606

3-Amathieu R et al, Difficult intubation in thyroid surgery: myth or reality? Anesth Anag. 2006 Oct;103(4):965-8

4- Meco et al Does ultrasonographic volume of the thyroid gland correlate with difficult intubation? An observational study. Braz J Anesthesiol. 2015 May-Jun;65(3):230-4

5-Khan et al The predictors of difficult tracheal intubations in patients undergoing thyroid surgery for euthyroid goitre. J Pak Med Assoc. 2010 Sep;60(9):736-8.

6-Patricia at al Annals of ORL 2015

7-Cheney FW. Changing trends in anaesthesia-related death and permanent brain damage. ASA Newsletter. 66(6),2002.

8- G. Frova, M. Sorbello Algorithmes for difficult airway management: a review. Minerva Anestesiologica 2009; 75: 201-9

Allegato 1 – Scala di Cormack e Lehane

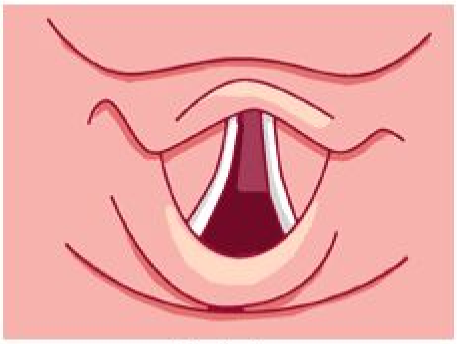
Grade 1

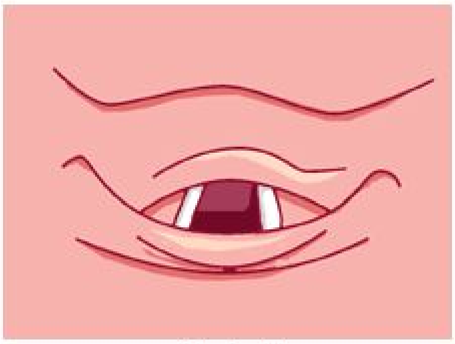
Grade 2

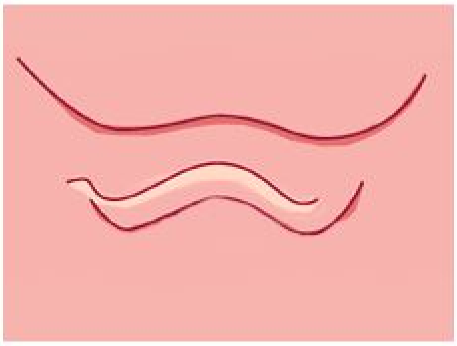
Grade 3

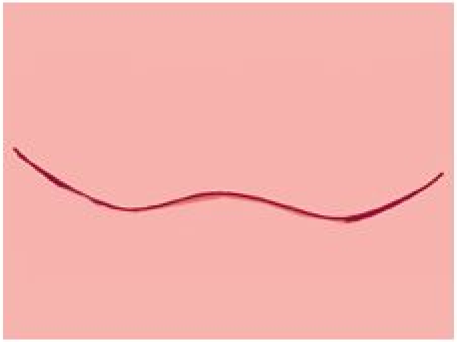
Grade 4

Grado 1. La maggior parte della glottide è visibile
Grado 2. Almeno la metà della glottide è visibile
Grado 3. Non è visibile la glottide, è visibile solo l’epiglottide
Grado 4. È visibile soltanto la base della lingua

Allegato 1 – Scala di Mallampati

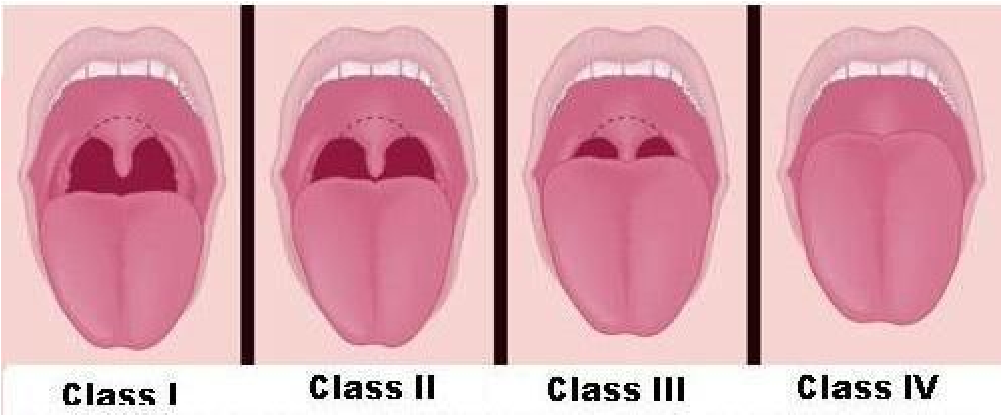

Classe I: palato molle, ugola e pilastri visibili.

Classe II: palato molle e ugola visibili.

Classe III: palato molle e base dell'ugola visibili.

Classe IV: solo il palato duro è visibile.
