# Supplementary material for: Prediction of difficult tracheal intubations in thyroid surgery. Predictive value of neck circumference to thyromental distance ratio
Source: PLoS One. 2019 Feb 27;14(2):e0212976. doi: 10.1371/journal.pone.0212976 (PMC6392301; doi:10.1371/journal.pone.0212976)
Supplement: S2 Protocol — Predictive value of neck circumference to thyromental distance ratio” study. (PDF) [file pone.0212976.s003.pdf]

Monocentric prospective observational study: Identification of predictive parameters of difficult intubation in patients undergoing thyroidectomy

Vers. 1.4

Protocol Version Date: 09/02/2017

Contents

1. General information
1. Background
1.1. Definitions
1.2. Problem size
1.3. Predictive parameters for difficult intubation
1.4. Predictive parameters for difficult intubation and thyroid pathology
1.5. Rationale of the study
2. Objectives of the study and hypothesis
    2.1. Primary objective and hypothesis
    2.2. Secondary objective
3. Inclusion and exclusion criteria
3.1. Enrollment
4. Materials and methods
4.1. Data collection
4.2. Statistical
4.2.1. sample size
4.2.2. Statistical analysis
5. Risks and benefits for the patient
6. Additional costs
7. Bibliography

1. General information

Title of the study

Identification of predictive parameters of difficult intubation in patients to be submitted to thyroidectomy

Protocol Version 1.4 - 09 February 2017

2. Background

2.1 Definitions

For difficult intubation [1], we mean a procedure that has been characterized by a difficult laryngoscopy or has required at least four attempts or more than five minutes for its execution regardless of the degree of experience of the anesthesiologist.

Difficult laryngoscopy refers to the impossibility of highlighting the glottis with the conventional curved blade laryngoscope. It corresponds to grades 3 and 4 of the classification of Cormack and Lehane (Annex 1) in which it is possible to highlight respectively only the epiglottis or the language alone.

2.2 Dimension of the problem

The incidence data reported by the literature on difficult airway control are often contradictory because they have suffered in the past from the lack of univocal definitions. 30% of all accidents related to anesthesia are sustained by difficulty in controlling the airways, 70% of these accidents resulted in death or permanent brain damage. The incidence of difficult intubation in the general population is between 0.5% and 20%.

2.3 Difficulty

prediction The prediction of difficulties is based on the objective examination of the patient with evaluation of the following parameters:

- measurement distance interdental
- test of Mallampati (Annex 2)
- visibility of pharyngeal structures
- measure of chin-thyroid distance
- measures maxillary prognathism
- global neck motility

The prediction of difficulty or inability to intubate is based on the modest deviation of several parameters or on the serious alteration of a single parameter. In particular, the literature [1] considers that the following parameters are

- distance <2 cm
- severe maxillary prognathism
- mallampati 4

- fixed neck in extension
- scarring or severe actinic effects on the lingual floor

2.3 Forecast of difficulties and thyroid pathology

Patients with thyroid disease represent a particular class of patients with regard to tracheal intubation due to the close anatomical contiguity between the thyroid and the trachea: it is therefore clear that an enlargement of the thyroid gland can cause dislocation and compression of the airways.

Studies have already been carried out on airways and thyroid pathology [2,3,4,5,6]. The percentage of difficult intubation was variable from 0% [4] to 12,9% [5] The criteria analyzed in literature are: interdental distance, migraine test, thyromentone distance, presence of receding chin, neck mobility, weight, age, presence of tracheal deviation to RX, presence of tracheal stricture at RX, malignant character of thyroid pathology, diameter of neck, presence of mediastinal goiter, glandular volume.

None of the studies in the literature has analyzed all these characteristics, but each has focused on different characteristics, thus leaving the lack of a study that globally analyzes these characteristics in this class of patients.

Furthermore, the conclusions are conflicting; characteristics such as opening of the mouth, moulding, thyromental distance, receding chin, neck mobility, tracheal deviation, malignant nature of thyroid pathology and tracheal stenosis have been significant in some studies and not significant in others; this finding can be justified by the low number of studies, in fact the highest number of studies is 326 patients [3] and the one with the lowest number of 50 patients [4].

2.5 Rationale of the study

The main strategy to avoid accidents of airway control is the planning and prediction of possible difficulties [7,8].

Although there are several scores on the prediction of difficulty for difficult intubation, there is little that exists regarding the class of patients with goiter [2,3,4,5,6].

The rationale is therefore to assess whether the predictive criteria of difficult intubation already validated for the population are also valid for the population suffering from thyroid disease, in addition to assessing whether there are peculiar characteristics of this population (neck diameter, tracheal displacement, mediastinal goiter) that can help in predicting the difficulty of intubation.

The study therefore aims to overcome the critical issues of previous studies both in terms of sample size, and as regards the overall analysis of parameters concerning difficult intubation without dwelling, unlike previous studies, only on some of them.

3. Objectives of the study and hypothesis

3.1. Primary objective and hypothesis

Evaluation incidence difficult intubation in the population with thyroid pathology compared to the data provided by the literature in the general population

3.2. Secondary objective

Identification of parameters or patterns of predictive parameters of difficult intubation during thyroidectomy.

The examination of NC/TMD for predicting DTI in patients undergoing thyroid surgery.

4. Inclusion / exclusion

criteria Inclusion criteria

- ✓ Surgical program of total or partial thyroidectomy
- ✓ Age> 18 years

Exclusion criteria

- ✓ Age <18 years
- ✓ Expected difficult intubation

4.1 Enrollment

This prospective observational study will be conducted in the operating rooms of the first and second floors of the Company's hospital Hospital of Padua.

All patients requiring thyroidectomy will be enrolled.

No clinical decision will be made based on the data collected for this study. In fact, during the study only information that is normally detected during a routine anesthetic visit will be recorded.

5. Materials and methods

During the routine anaesthesiology visit, upon collection of the consent, the following parameters will be recorded on the data sheet.

- Opening mouth (cm)
- Distance thyreomental (cm)
- Test of Mallampati (Annex 2)
- Mobility of the neck (<80 °; 80-90 °;> 90 °)
- Inability to prognath
- Body weight (kg)
- History of previous intubation difficult
- Tracheal deviation to the chest radiography
- Diameter of the goiter (cm)
- Presence of mediastinal goiter
- Benign or malignant nature of the thyroid pathology

On the day of the surgical intervention after tracheal gold intubation the presence or absence of difficulty in the maneuver will be reported on the data sheet ; in particular:

- Cormack Scale (Annex 1)
- Number of attempts necessary to successfully perform the intubation
- Time elapsed since the induction of general anesthesia and intubation successfully
- If advanced airway instrument were used and which ones

5.1 Data collection

Data will be collected in a data collection form. Patients will be assigned with a numerical code to facilitate subsequent processing; the data will be stored in an anonymised form.

5.2 Statistics

5.2.1 Sample Size

The estimates of study power and sample size were based on an assumption of a difference of 4% in the incidence of difficult intubation between the general population of patients undergoing surgery
[1] and the population of patients undergoing thyroid surgery [4], We use an α-value of 5%, a power
(1-β error) of 95%, a drop out percentage of 5%. The results of the analysis indicated that a sample size of 515 patients was necessary to estimate the prevalence of DTI in a population of patients undergoing thyroid surgery.

The data for each continuous variable will be analyzed for a normal distribution using the Kolmogorov-Smirnov test. Results for continuous variables with normal distributions will be expressed as mean (standard deviation) values; those with non-normal distributions will be expressed as median (interquartile range) values. Analysis of data with a normal or a non-normal distribution will be performed using the two-tail Student’s t-test and the Mann-Whitney U test, respectively. The results for analyses of categorical variables will be reported as numbers (percentages) and were compared between groups using Chi-square tests. To determine the strength and direction of association between two variables, we will use the Bravais-Pearson’s correlation test for variables with a normal distribution and Spearman’s rank correlation test for variables that will not meet the assumptions of a normal distribution.

To determine the relationships between the dependent categorical variable (i.e., DTI) and one or more independent categorical variables (i.e., DTI predictors), we will perform a multiple logistic regression analysis to calculate odds ratios (ORs) with 95% confidence intervals (CIs).All statistical analyses will be performed using R version 3.4.0 (2017-04-21). P-values<0.05 will be considered to indicate a statistically significant result.

6. Risks and benefits for the patient

The study is prospective observational. No clinical decision will be made based on the collected data. There are no direct benefits or risks added to those of a standardized clinical procedure.

7. Additional costs

The study is prospective and observational study without additional costs.

8. Limitations of the Study

The main limitation of the study is its monocentric nature.

8.Bibliography

1-Recommendations for airway control and difficulty management. SIAARTI Study Group Coordinator Flavia Petrini Minerva Anestesiologica 2005; 71: 617-67

2-Bouaggad A et al, Prediction of the difficult tracheal intubation in thyroid surgery. Anesthesia & Analgesia: August 2004 - Volume 99 - Issue 2 - pp 603-606

3-Amathieu R et al, Difficult intubation in thyroid surgery: myth or reality? Anesth Anag. 2006 Oct; 103 (4): 965-8

4- Meco et al Does the ultrasonographic volume of the thyroid gland correlate with difficult intubation? An observational study. Braz J Anesthesiol. 2015 May-Jun; 65 (3): 230-4

5-Khan et al The predictors of difficult tracheal intubations in patients undergoing thyroid surgery for euthyroid goitre. J Pak Med Assoc. 2010 Sep; 60 (9): 736-8.

6-Patricia at the Annals of ORL 2015

7-Cheney FW. Changing trends in anaesthesia-related death and permanent brain damage. ASA Newsletter. 66 (6), 2002.

8- G. Frova, M. Sorbello Algorithmes for difficult airway management: a review. Minerva Anestesiologica 2009; 75: 201-9

Annex 1 - Scale of Cormack and Lehane

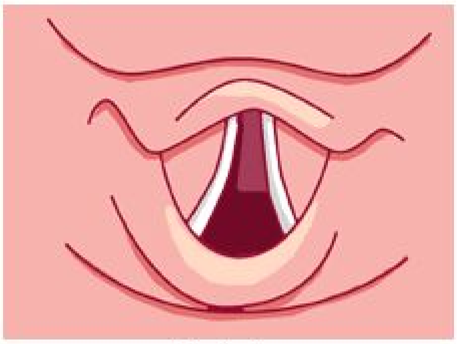
Grade 1

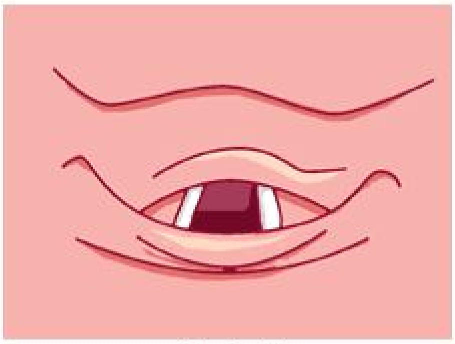
Grade 2

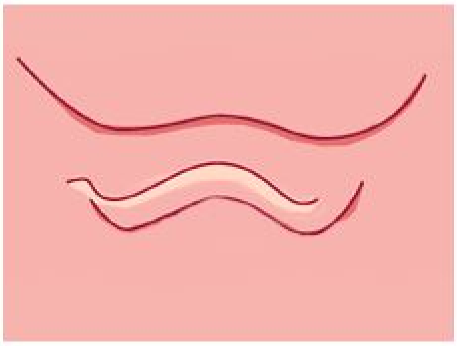
Grade 3

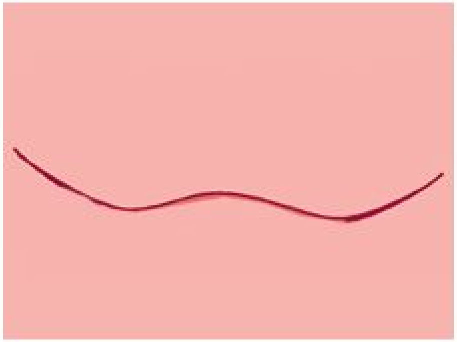
Grade 4

Grade 1. Most of the glottis is visible
Grade 2. At least half of the glottis is visible
Grade 3. The glottis is not visible, only the epiglottis
Grade 4 is visible. language base

Annex 1 - Mallampatiscale

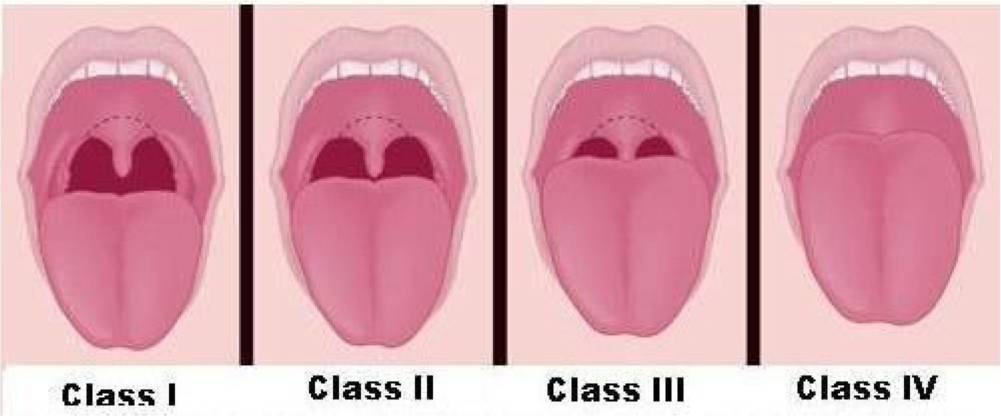

Class I: soft palate, uvula and visible pillars.

Class II: visible palate and uvula.

Class III: soft palate and visible uvula base.

Class IV: only the hard palate is visible.
